# Do pediatric emergency departments pose a risk of infection?

**DOI:** 10.1186/1471-2431-11-2

**Published:** 2011-01-07

**Authors:** Caroline Quach, Dorothy Moore, Francine Ducharme, Dominic Chalut

**Affiliations:** 1 Infectious Diseases Division, Department of Pediatrics, The Montreal Children's Hospital, McGill University Health Center, (2300 Tupper Street), Montreal, (H3H 1P3), Canada; 2 Department of Pediatrics, CHU Sainte-Justine, (3175 Côte Ste-Catherine), Montreal, (H3T 1C5), Canada; 3 Division of Emergency Medicine, Department of Pediatrics, The Montreal Children's Hospital, McGill University Health Center, (2300 Tupper Street), Montreal, (H3H 1P3), Canada

## Abstract

**Background:**

There is no data documenting the existence of a risk of infection transmission in ambulatory healthcare settings but concern remains. Our objective was to determine the risk of infection associated to a pediatric Emergency Department (ED) visit and the predictors of infection in children aged 5 years and less.

**Methods:**

Children aged 5 years and less with an ED visit between February and April of a non pandemic season were recruited and followed-up by telephone interviews to ascertain the development of new respiratory and gastrointestinal infections. Approximately half of the parents were called 7-10 days after their child's ED visit. The other half were called at least 14 days after the visit and served as the ED-unexposed group. The principal outcome was the onset of a new infection in the week preceding the phone interview, using standardized definitions. Proportions of children with new infections were calculated in both groups and logistic regression was used to adjust for potential confounders.

**Results:**

A total of 304 children (mean age 2.4 years) were followed. Of the 137 children with a recent ED visit, 21 (15.3%) developed an infection compared to 39 of 167 (23.4%) of those without a recent visit. The relative risk (RR) associated with ED exposure was 0.7 (95%CI 0.4-1.1). As 85 children with a recent ED visit presented to the ED with a viral infection, we repeated the analysis excluding them to improve our capacity to detect new infections: 9 children (17.3%) developed an infection (RR = 0.7 [95%CI 0.4-1.4]). The only factor associated with an increased risk of infection was an intra-familial infectious contact (RR 9.9; 95%CI 1.7-58.9).

**Conclusion:**

A visit to a pediatric ED does not result in a detectable increased risk of infection above the risk in the community. This is likely explained by the high baseline risk of infections in young children. However, we cannot eliminate the possibility that a risk of infection may still exist and would warrant a larger study to document.

## Background

Young children readily acquire and transmit infections. A pre-school-aged child will, on average, contract 8.3 colds per year [1]. Children frequently harbor infectious organisms and may shed pathogens, especially respiratory and gastrointestinal viruses, even if asymptomatic. In places where young children gather, such as daycares and potentially ambulatory settings waiting areas, close proximity of large numbers of infectious and susceptible hosts favors transmission. Behavioral characteristics such as incontinence or inadequate hygiene, frequent mouthing of hands and objects including toys, drooling, and direct contact between children during play facilitate spread of infection.

Healthcare-associated infections (HAI) represent an important risk for the health of hospitalized patients. In pediatrics, studies have demonstrated that the majority of HAIs acquired on general pediatric wards were viral gastroenteritis (55%) and respiratory tract infections (22%), showing the importance of viruses as healthcare-associated pathogens in this particular patient population [2,3]. These viruses are quite prevalent in the community and are introduced in the hospital setting by admitted patients or their families. In hospital, HAI surveillance programs use standard definitions and procedures [4]. However, there is to our knowledge no data evaluating the risk of healthcare-associated infection following an emergency department (ED) visit, which differs from hospitalization as the duration of contact with other patients is usually shorter but the density and number of patients with whom there could be a contact is much higher, particularly in the waiting area where an important proportion of patients are consulting for infectious diseases.

ED has been perceived as a potential source of infection and these settings have been urged to implement source containment measures to prevent transmission of respiratory pathogens. However, these measures are a challenge to implement, especially in pediatrics because of the inability of children to comply with the recommended measures. The objective of the study was thus to evaluate the risk of infection associated with a visit to a pediatric emergency department and the predictors of infection in children aged 5 years and less.

## Methods

### Study Setting

The Montreal Children's Hospital is a pediatric teaching hospital with a medical and surgical ED that serves the Island of Montreal and its surroundings. During the winter months (January to March), an average of 6000 children are seen per month with approximately 4000 aged 5 years and less. Approximately 50% of these children consult because of a respiratory illness. The average waiting time during the winter months is 2 hours between registration and physician encounter, usually spent in the waiting area. The total time spent in the ED between registration and discharge is on average 5 hours. Our ED had a common waiting area measuring 2500 square feet that could accommodate up to 80 people. There were no available toys but play modules that were cleaned daily. The total surface of the ED was 7000 square feet, comprising 16 examination rooms: 2 of which were isolation rooms, 12 stretchers and 10 chairs for asthmatic patients' treatment. This study was approved by The Montreal Children's Hospital Research Ethics Board.

### Study Population

Children aged 5 years and less seen at the MCH ED between February and April of a non-pandemic season were eligible to participate. This time-period was selected, as it is the peak season of respiratory and gastroenteritis viral infections. As this was a pilot study done to provide baseline data for further studies, consent to call was obtained from a convenience sample of children during their ED visit. Each child participated only once in the study.

**Figure 1:**
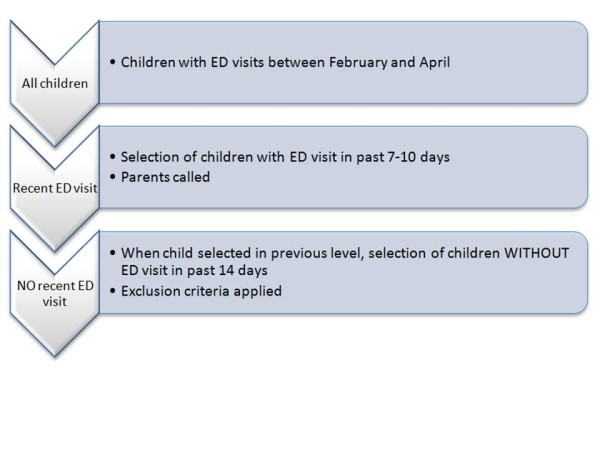
**Logistics of study**.

A prospective cohort of children with an ED visit was created (figure 1). Approximately half of the parents were called 7-10 days after their child's ED visit. The other half were called at least 14 days after the visit and served as the group with no recent ED visit. All parents were called during the months of March and April. Given that the average incubation period for most common viral respiratory tract and gastrointestinal infections ranges from 2 to 6 days [5], we elected to conduct phone interviews 7-10 days following the ED visit, allowing us to capture most infections associated with the ED visit.

Two carefully trained research assistants conducted the phone interviews using a standardized questionnaire and a standardized case report form. The duration of a phone interview was between 5 to 10 minutes. All parents reached agreed to answer.

Children were excluded if they were hospitalized in the 2 weeks prior to the phone interview and if they had any medical condition leading to significant immunosuppression such as HIV, cancer with chemotherapy, congenital immunodeficiency, or if they were taking immunosuppressive drugs. As viral co-infections may occur, we did not exclude patients who presented to the ED with viral infections.

### Definition of outcome

The primary outcome of interest was the development of a new acute respiratory or gastrointestinal tract infection in the at-risk period. Standardized surveillance definitions for respiratory and gastrointestinal infections used for healthcare-associated infections surveillance were used [6].

Infection associated with an ED visit: A new infection was defined as a *new *onset of any of the following clinical manifestations, 3 days or more after the ED visit. If symptoms of infection were present on the day of the visit, we considered it as a new episode after an interval of 3 days without the symptom. In summary, a respiratory tract infection was identified if any of the following sign or symptom was present: Rhinorrhea or congestion, cough in a non-asthmatic, hoarse voice, or sore throat. If a chronic underlying respiratory condition existed: there needed to be a change in the quality or quantity of respiratory secretions, or a diagnosis of a respiratory tract infection made by a physician. Gastroenteritis was identified in the presence of watery stool and increase in stool frequency for 24 hours or more in the absence of another identifiable cause (antibiotics, dietary changes).

The following time periods were used for surveillance of new symptoms. For children with a recent ED visit, symptoms were sought going forward from the 3^rd ^to the 7^th ^day after the ED visit or in other words, in the period extending from 4 days before until the day of the phone interview if the latter was done on day 7 after the ED visit; or between the 7^th ^and 3^rd ^day before the phone interview, if it was conducted 10 days after the ED visit. To make the at-risk period comparable in both groups, symptoms were sought going backwards during the 4 days prior to the phone interview in children without a recent ED visit.

### Data collected

Data collected were: patient's demographic data (age and sex from ED database) and daycare attendance, onset of new symptoms of infection starting in the four days prior to the phone interview in the child or other family members, age of siblings and their daycare attendance, passive smoking, past medical history including vaccination history. We also asked if the child had any additional contact with the healthcare system in the preceding 7 to 10 days (Emergency Department, clinic, or local clinics). The ED database was used to obtain the physician's discharge diagnosis, as well as the registration, consultation, and discharge times and dates.

### Sample size

As this was a pilot study, we aimed for a total sample size of 300, based on the previous study from Lobovitz et al [7]. Assuming a baseline risk of infection of 30%, this sample size would have given us an 80% power to detect an increase in 50% of the risk of infection following an ED visit.

### Statistical Analysis

Descriptive statistics (mean, standard deviation), κ^2 ^tests, and Student *t *test were used for univariable analyses. The incidence of new infections was calculated in both groups as well as the relative risk (RR) and the attributable risk associated with an ED visit. Factors associated with the onset of a new infection were analyzed using logistic regression in a stepwise fashion. Variables were kept in the final model if their coefficient was statistically significant, if they confounded variables already present in the model (change in 10% of the coefficient) or if they improved significantly the model fit. All *p *values were considered significant at 0.05 and were 2-sided (SAS Institute, version 9.2, Cary, NC). A sensitivity analysis excluding children with a recent ED visit who presented to the ED with an infection was also done as the presence of an infection at the time of the visit could have decreased our capacity to detect the occurrence of new symptoms.

## Results

Of the 320 signed consent forms received from the ED, we reached and interviewed parents of 304 children (95%) who visited the MCH ED between the months of February and April of a non-pandemic season. Phone interviews were done during the months of March and April. Of the 304 children, 137 (45.1%) had a recent ED visit and 167 had a remote ED visit. The mean age of the total group was 2.4 years (median 2.1, standard deviation [SD] 1.6). There was no significant difference in mean age or sex distribution between the two groups (table 1).

**Table 1:** Baseline characteristics of the cohort

Characteristics	Recent ED visit	No recent ED visit	p-value
Number	137	167	
Age in years (mean ± SD)	2.3 ± 1.6	2.4 ± 1.6	NS
Sex: Male (%)	66 (48)	92 (55)	NS
≥ 1 dose of influenza vaccine (%)	42 (30.6)	51 (30.5)	NS
Daycare attendance (%)	77 (56.2)	99 (59.3)	NS
At least one sibling (%)	88 (64.2)	103 (61.7)	NS
At least one sibling in daycare (%)	24 (17.5)	36 (21.6)	NS
Exposed to passive smoking (%)	20 (14.6)	20 (12)	NS
Intrafamilial infectious contact (%)	2 (1.5)	6 (3.6)	NS

ED: Emergency Department

### Risk of infection following ED visit

Overall, 12 children developed a gastroenteritis (4%), 44 children a respiratory infection (14.5%), and 7 children developed a new fever (2.3%) - 3 in children with other infectious symptoms - during the surveillance period for a total of 60 children with a new infection (19.7%). Table 2 breaks down these infections by ED exposure status. As shown in the table, recent ED visit did not seem associated with an increased risk of a new infection. Of the 60 children who developed a new infection, 8 (13.3%) had an intra-familial infectious contact compared to none of the 244 children who did not develop a new infection during the surveillance period. Similarly, 4 of the 60 children (6.7%) who developed a new infection visited another clinic in the week prior to the phone interview for reasons other than infections compared to 1 of 244 children without a new infection (0.4%).

**Table 2:** New infections developing in children during the surveillance period by ED exposure category

Outcome	Recent ED visit	No recent ED visit	RR (95%CI)
		
	n = 137	n = 167	
GE	4 (2.9%)	8 (4.8%)	0.6 (0.2-2.0)
RTI	13 (9.5%)	31 (18.6%)	0.5 (0.3-0.9)
Any infection	21 (15.3%)	39 (23.4%)	0.7 (0.4-1.1)

GE: Gastroenteritis; RTI: respiratory tract infection; ED: Emergency Department

The multivariable analysis where all children were analyzed (n = 304) did not reveal any significant risk factor predicting the acquisition of any new infection. All the characteristics presented in table 1 were used. The odds-ratio (OR) associated with a recent ED visit was 0.6 (95%CI 0.3-1.1). No confounding variables were identified. On the other hand, the risk of developing a new respiratory infection was lower among those with a recent ED visit (OR 0.5; 95%CI 0.2-0.9). Having an intra-familial infectious contact was an independent risk factor for acquiring a new respiratory infection during the surveillance period (OR 18.8; 95%CI 3.6-98.4).

As 62% of the children with a recent ED visit presented to the ED with symptoms of viral infection, we performed a sensitivity analysis excluding them as potential difficulty may arise when trying to differentiate signs of a new infection at the same site i.e. respiratory or gastrointestinal in a child who was already symptomatic, because of overlapping symptomatic periods. As shown in table 3, the percentage of new infections in children with recent ED visits was slightly higher when only previously non-infected children were compared. We then repeated the multivariable analysis comparing children with a recent ED visit but without any prior infection to children without a recent ED visit. There was no statistically significant risk associated with ED exposure (OR 0.7; 95%CI 0.3-1.6). The same sensitivity analysis was repeated looking at the development of a new respiratory infection. There was no difference in the risk of developing a new respiratory infection among those with and without ED visits (OR 0.8; 95%CI 0.3-1.9) but having an intra-familial infectious contact remained a risk factor for the development of a new respiratory infection during the surveillance period (OR 9.9; 95%CI 1.7-56.9).

**Table 3:** New infections developing in children during the surveillance period by ED exposure category (Children without prior infection only)

Outcome	Recent ED visit	No recent ED visit	RR (95%CI)
		
	n = 52	n = 167	
GE	0 (0%)	8 (4.8%)	
RTI	7(13.5%)	31 (18.6%)	0.7 (0.3-1.6)
Any infection	9 (17.3%)	39 (23.4%)	0.7 (0.4-1.4)

GE: Gastroenteritis; RTI: Respiratory tract infection; ED: Emergency Department

### Children with recent ED visits

The 137 children in this cohort waited an average (± SD) of 1.9 hours ± 1.9 - median 1.3 hours - between registration and their first encounter with the physician. According to the Canadian Triage and Acuity Scale (CTAS) [8], the majority of children were triaged in categories 3 (26%), and 4 or 5 (32%). The average length of stay (± SD) in the ED - between registration and discharge - was 3.4 hours ± 3.0, with a median of 2.3 hours. Eighty-five children (62%) came to the ED with an infection. Seventeen (12%) presented to the ED with fever, 5 (4%) with diarrhea, 19 (14%) with a runny nose, 8 (6%) with a blocked nose, and 20 (15%) with cough. When asked about infection prevention measures, 61% of parents said that they were able to find tissues when needed, 68% found hand-washing material when needed, and 80% noticed posters promoting hand washing.

## Discussion

To our knowledge, this study is the first looking at the risk of acquisition of an infection associated with an ED visit. There was no increased risk of infection in children with a recent pediatric ED visit when compared to those without. This remained even when only children who came to the ED for non-infectious causes were analyzed. We performed this sensitivity analysis because of the potential difficulty that may arise when trying to differentiate signs of a new infection at the same site i.e. respiratory or gastrointestinal in a child who was already symptomatic. Overlapping symptomatic periods could have biased our results in favor of a lower rate of infections among children with a recent ED visit. The OR in both analyses were similar with overlapping confidence intervals. These findings are likely to be generalized to other ED in North America, as the population seen and the average waiting time are similar to other facilities [9]

When looking at the risk of acquiring respiratory infection following an ED visit, it was surprising to see that children with a recent visit were less likely than those without a recent ED visit to develop a new respiratory infection during the surveillance period. This decreased risk did not remain statistically significant when looking only at previously uninfected children, likely because of the previously mentioned potential bias. However, one possible explanation for the trend towards decreased risk could be a lower daycare attendance in the few days surrounding the ED visit for those acutely ill. This data was not available but would be interesting to assess. The most important risk factor for developing a new respiratory infection was an intra-familial infectious contact.

Our findings are in keeping with a very small amount of literature on the risk of infection in ambulatory care settings. Lobovits et al. [7], showed that there was no infectious risk associated to an office visit. The authors identified symptoms compatible with a respiratory or gastrointestinal infection in 30% and 32% of children with and without a recent clinic visit respectively. However, in this office practice, well-child visits were scheduled separately from visits for illness and patients were rarely in the waiting room for longer than 20 to 30 minutes.

In the absence of literature in ambulatory care settings, we reviewed the literature on daycare settings, as both settings are somewhat analogous as they provide close contact between numerous children, many of whom with symptoms of infection. Studies have shown an increased risk of infection associated with daycare attendance, when compared to children cared for at home [10-12]. Similar results were found in a birth cohort where the risk of respiratory infection was 2.7 times higher for children in daycare compared to children who stayed home. This study also showed that having siblings increased the risk of infection (OR 2.6; 95%CI 2.0-3.4) [13]. From these studies, it may be concluded that the greater the number of contacts - and potentially the length of contact - the higher the risk of infection. However, an ED visit differs from daycare attendance: an ED visit usually occurs only once, in opposition to daycare and children coming to the ED are usually sicker.

Currently, ED waiting rooms are providing masks, tissues and alcohol-based hand-rinse in their waiting areas. However, the pediatric setting poses additional challenges above and beyond compliance and crowding issues that will also occur in adult settings. Although tissues and masks are available in our ED waiting room, it is difficult for parents to always comply with recommended measures. Moreover, alcohol-based hand-rinse is more challenging to use in children, as they pose a risk of ingestion. The hand-rinse is also difficult to apply to young children's hands. In our institution, the Infection Control Committee had therefore recommended the use of individually wrapped hand-wipes as well as making alcohol hand-rinses available.

During this study, the infection prevention measures available in our ED waiting area were availability of tissues and posters promoting hand hygiene. Provision of alcohol-free hand wipes was in the process of being implemented and alcohol-based hand gel was not available in patient-care area because of the risk of ingestion.

Our study has some limitation. As this was a pilot study, our study procedure did not allow us to keep track of the number of patients approached. Sheets explaining the study procedure with consent to be called were left in examination rooms by clerks upon setting up between patients, completed by parents while the child waited to be seen, and picked up by clerks upon patient's discharge. It is thus possible that the patients recruited in this study were a biased sample of the overall patient population seen in our ED, as it was much more likely for parents coming for more benign reasons to fill out a consent form. However, these patients represent the majority of patients seen in pediatric EDs and were the main target of our study and should be representative of the population seen in other ED [6]. Given the baseline high rate of infection, our small sample size may not have allowed us to detect a difference in the risk of infections among children with and without recent ED visits. If we take the sub-sample of children who did not come to the ED for an infection, the upper limit of our RR 95%CI for the risk of developing any infection following an ED visit may be translated in a maximal risk of infection, based on our study population, of 30% of children with compared to 21.9% of children without a recent ED visit, a proportion closer to that found by Lobovits et al. [7]. One of our study's strength was the use of standardized and validated definitions for the diagnosis of respiratory and gastrointestinal infections.

## Conclusion

In conclusion, based on this small study, a visit to a pediatric ED does not seem to result in a detectable risk of infection - either respiratory or gastrointestinal - above the risk in the community, even without using additional precautions. This is likely because of a high baseline risk of infection in this particular population - either because of daycare attendance or intra-familial infection. However, we cannot eliminate the possibility that a risk of infection may still exist and would warrant a larger study in a different population where the risk of respiratory or gastrointestinal infection in patients without an ED visit is low, such as long-term care facilities or nursing homes residents.

## List of abbreviations used

CI: Confidence interval; ED: Emergency Department; HAI: Healthcare-associated infection; OR: Odds ratio; RR: Relative risk; SD: Standard deviation

## Competing interests

The authors declare that they have no competing interests.

## Authors' contributions

CQ designed the protocol, supervised the data collection and data entry, analyzed the data, drafted the manuscript; DM contributed to the protocol design and critically reviewed the manuscript; FD contributed to the protocol design and critically reviewed the manuscript; DC helped in participants' enrolment and critically reviewed the manuscript. All authors have read and approved the manuscript.

## Pre-publication history

The pre-publication history for this paper can be accessed here:

http://www.biomedcentral.com/1471-2431/11/2/prepub
